# Hydroxychloroquine improves pregnancy outcomes of women with positive antinuclear antibody spectrum test results

**DOI:** 10.3389/fmed.2023.1113127

**Published:** 2023-04-24

**Authors:** Shenglong Ye, Yuanying Liu, Xueqing Zhao, Yue Ma, Yongqing Wang

**Affiliations:** Department of Obstetrics and Gynecology, Peking University Third Hospital, Beijing, China

**Keywords:** hydroxychloroquine, pregnancy outcomes, antinuclear antibodies, placental function, retrospective study

## Abstract

**Background:**

Empirical use of Hydroxychloroquine (HCQ) in patients with positive antinuclear antibody spectrum (ANAs) test result is controversial regarding its impact on improving perinatal outcomes. This study aimed to investigate the effect of HCQ on adverse pregnancy outcomes associated with placental dysfunction in ANAs-positive patients.

**Methods:**

The study included pregnant women with positive ANAs test result from 2016 to 2020 in our center, and divided into a weakly positive and a positive group in just ANA positive patients among them. Univariate and multivariate analyses were conducted to determine the effect of HCQ on pregnancy outcomes in each subgroup. Stratified and interactive analyses were performed to assess the value of HCQ in improving pregnancy outcomes.

**Results:**

(i) A total of 261 cases were included, accounting for 30.60% of pregnancy complicated with autoimmune abnormalities, and 65.12% of them used HCQ during pregnancy. (ii) The application of HCQ significantly reduced the incidence of early-onset preeclampsia (1.18% vs. 12.09%, *p* = 0.040) and small-for-gestational-age infants (10.06% vs. 25.84%, *p* = 0.003) in the ANAs-positive population, increased birth weight (3075.87 ± 603.91 g vs. 2847.53 ± 773.73 g, *p* = 0.025), and prolonged gestation (38.43 ± 2.31 vs. 36.34 ± 5.45 weeks, *p* < 0.001). (iii) A total of 185 just ANA-positive patients were stratified according to titers. Among them, the rate of HCQ usage was significantly higher than that in the weakly positive group (81.03% vs. 58.27%, *p* = 0.003). (vi) Stratified univariate analysis showed that HCQ usage in the ANA-positive group could reduce the incidence of preeclampsia (2.13% vs. 27.27%, *p* = 0.019) and prolong gestation (38.29 ± 2.54 vs. 34.48 ± 7.68 weeks, *p* = 0.006). In the ANA-weakly positive group, HCQ significantly reduced the incidence of preeclampsia (6.76% vs. 28.30%, *p* = 0.002), early-onset preeclampsia (1.35% vs. 13.21%, *p* = 0.027), and small-for-gestational-age infants (7.89% vs. 35.19%, *p* < 0.001). Multivariate regression analysis showed that HCQ significantly reduced the incidence of preeclampsia in both groups. Intergroup interaction analysis showed no significant difference in the value of HCQ in reducing the incidence of preeclampsia between the two groups.

**Conclusion:**

ANAs positivity is an important abnormal autoimmunity type in pregnancy. HCQ can be considered as a choice for improving adverse pregnancy outcomes related to placental dysfunction, such as preeclampsia, in this population.

## 1. Introduction

Antinuclear antibody (ANA) are auto-antibody that target various eukaryotic cell components, and ANA spectrum (ANAs) are commonly utilized in clinical practice as a crucial biological marker of autoimmune diseases (AID) (1). ANAs positivity has been observed at a higher detection rate in populations with infertility and recurrent miscarriage. However, the mechanism by which ANAs contribute to adverse pregnancy outcomes remains uncertain (2–4).

Hydroxychloroquine (HCQ) has emerged as a essential therapeutic option for pregnant patients with systemic lupus erythematosus (SLE), Sjögren’s syndrome syndrome (SS), and undifferentiated connective tissue disease (UCTD), owing to its multilevel immune anti-inflammatory effect (5). Prior studies have demonstrated its efficacy in improving perinatal outcomes in the aforementioned disease population, particularly in reducing the incidence of preeclampsia and other adverse pregnancy outcomes (6, 7). Despite these promising results, there is ongoing debate surrounding the application of HCQ in ANAs-positive populations. Given its pharmacological mechanism, post-pregnancy immune status, coagulation function changes, and pathogenesis of placental dysfunction-related diseases, we aimed to investigate the potential benefit of HCQ in mitigating adverse pregnancy outcomes associated with placental dysfunction in pregnant patients with ANAs positivity. Our study analyzed cases of pregnant patients with ANAs positivity, and evaluated the impact of HCQ on disease prevention and treatment, specifically preeclampsia.

## 2. Materials and methods

### 2.1. Inclusion criteria

This research was a retrospective cohort study conducted at a single medical center. The study enrolled all pregnant patients with ANAs positivity who were admitted to the obstetric department of Peking University Third Hospital between January 1, 2016, and December 31, 2020. The study utilized hospitalization and delivery information obtained from complete patient case files, and ensured the availability of unambiguous diagnostic information.

### 2.2. Exclusion criteria

The exclusion criteria for this observational and retrospective cohort study were defined as follows: (i) pregnancy loss before 14 weeks of gestation; (ii) incomplete case information due to delivery at a hospital other than the Peking University Third Hospital or other reasons; (iii) abortion or midterm induction of labor not related to AID condition factors, where the mother and fetus remained stable during pregnancy, but the pregnancy was terminated due to concerns about the potential risk of combined AID, the detection of fetal malformations, or social factors.

### 2.3. Case screening process

The study population comprised individuals who tested positive for ANAs, but who lacked clinical symptoms, medical history, or signs of AID. Participants did not meet any diagnostic criteria for AID and did not test positive for any other autoantibodies. Given the potential for overdiagnosis or inaccurate detection of autoimmune abnormalities, we employed a preliminary screening process that encompassed all cases possibly related to such abnormalities, followed by rigorous diagnostic re-evaluation to minimize the risk of omissions.

During the designated period, we conducted an initial screening of patients in the electronic medical record system using relevant diagnostic keywords and ICD coding standard diagnostic terms. Specifically, we used terms such as “autoimmune disease,” “connective tissue disease,” “autoantibody-positive,” “ANA or ANA spectrum positive,” “adverse pregnancy and delivery history,” and “recurrent spontaneous abortion, recurrent miscarriage, or habitual abortion” to identify potential cases for inclusion in our study population.

Subsequently, the medical records of the eligible patients were scrutinized, and their diagnostic records were analyzed to evaluate their autoimmune symptoms, physical examinations, and auxiliary examinations. We meticulously evaluated each case and ruled out co-occurring positive autoantibodies or definite diagnoses of AID. We also recorded the highest titer values for each case.

### 2.4. Classification, grouping, and stratification standards

The present study aimed to investigate the effects of HCQ application during pregnancy in pregnant women with positive ANAs test results. The study population was divided into two groups: the exposed group (study group), which consisted of pregnant women who received HCQ treatment during pregnancy, and the non-exposed group (control group), which consisted of pregnant women who did not receive HCQ treatment during pregnancy. The duration of HCQ dosing was defined as taking hydroxychloroquine sulfate at a dose of 0.1–0.2 g/dose, 2 times/day, for at least 1 month. Additionally, ANA-positive patients were further classified into two groups based on their ANA titres: weakly positive (ANA levels ≤1:80) and positive (ANA levels >1:80).

### 2.5. Observation indicators and outcome indicators

(i) The study collected demographic and medical history information, including age, gravidity times, previous deliveries, previous early pregnancy loss (spontaneous abortion or embryo arrest), obesity, and history of preeclampsia. (ii) Current pregnancy indicators were considered, such as weight gain during pregnancy, whether assisted reproduction was used, multiple pregnancies, combined kidney disease, combined chronic hypertension, pregestational diabetes mellitus, gestational diabetes mellitus, preeclampsia, and early onset preeclampsia. (iii) Pregnancy outcomes were assessed and included the week of delivery, the mode of delivery, neonatal birth weight, the neonatal Apgar score, small for gestational age (SGA), and pregnancy loss in mid-to-late pregnancy (embryonic abortion at more than 14 weeks of gestation, missed abortion, or stillbirth). (iv) Medication use during pregnancy was defined, including the use of HCQ (duration and indications), prophylactic anticoagulation (aspirin and/or low molecular heparin), or other immune anti-inflammatory therapies (immunosuppressive agents and/or glucocorticoids).

The study aimed to investigate the effects of HCQ use during pregnancy on maternal and neonatal outcomes related to placental function. The primary outcomes were preeclampsia, early-onset preeclampsia, and SGA, while the secondary outcomes included the gestational week of delivery, birth weight, neonatal asphyxia, and mid-to-late pregnancy loss.

- Obesity was defined as an adult body mass index ≥28 kg/m^2^ before pregnancy.
- Neonatal asphyxia was defined as a neonatal Apgar score of ≤7 at the first minute, reflecting the intrauterine condition of the fetus. The analysis of neonatal asphyxia in this study was performed on live-born babies (neonates, viable infants) at all gestational weeks.
- The present study involved the recording of birth weight for fetuses delivered at ≥24 weeks of gestation. A semicustomized fetal growth curve was developed by referring to the 2019 Expert Consensus on Fetal Growth Restriction, calibrated to the Chinese population (8). Birth weight was defined as SGA if it was below the 10th percentile of the desirable weight for the same gestational age. The analysis of birth weight and SGA was limited to fetuses delivered at ≥24 weeks of gestation, whether they were live-born, aborted or stillborn.
- Preeclampsia cases were defined in accordance with national and international guidelines. Early-onset preeclampsia referred to the termination of pregnancy before 34 weeks of gestation due to preeclampsia (9).

### 2.6. Statistical analysis of data

The data were collected and organized using Microsoft Excel 2020, and statistical analysis was conducted using SPSS 26.0 software.

For measurement data, the mean ± standard deviation was used depending on the normality test results. Independent sample *t*-tests, one-way ANOVA or nonparametric tests were employed for comparison between groups, and linear regression was used for multifactor analysis.

Count data were presented as the number of cases (percentage), and the chi-square test was used for group comparison. Multifactor analysis was performed using logistic regression. A two-sided *p* < 0.05 was considered statistically significant for all tests.

### 2.7. Ethics statement

The present study received ethical approval from the Institutional Review Board and Ethics Committee of the Third Hospital of Peking University, People’s Republic of China (2022 No. 203‑01).

## 3. Results

### 3.1. Demographic and clinical characteristics of pregnant women with a positive ANAs test result

A total of 261 pregnant patients with ANAs positivity were enrolled, representing 30.60% (261/853) of pregnant patients with autoimmune abnormalities during the same period. Among the ANAs positive patients, 65.12% received treatment with HCQ during pregnancy. The demographic information and clinical characteristics of the ANAs positive and negative groups are presented in Table 1.

**Table 1:** Demographic information and clinical characteristics of antinuclear antibody spectrum-positive population.

	Application of HCQ	Without HCQ	*t/X^2^*	*p-*value
	170 (65.13)	91 (34.87)		
Demographic and medical history information			
Age (years)	33.81 ± 3.51	34.55 ± 4.82	1.230	0.196
Gravidity (times)	2.80 ± 1.17	2.52 ± 1.40	1.640	0.103
Primiparas [*n* (%)]	149 (87.65)	64 (70.33)	10.718	0.001
History of early pregnancy loss ≥2 times [*n* (%)]	65 (38.24)	20 (21.98)	7.113	0.008
Obesity [*n* (%)]	6 (3.53)	12 (13.19)	8.609	0.003
History of pre-eclampsia [*n* (%)]	6 (3.53)	8 (8.79)	2.279	0.131
This pregnancy			
Embryo transfer [*n* (%)]	35 (20.59)	20 (21.98)	0.069	0.793
Multiple pregnancy [*n* (%)]	9 (5.29)	4 (4.40)	<0.001	0.984
Weight gain during pregnancy (g)	12.42 ± 4.87	11.61 ± 4.61	1.324	0.187
Combined kidney disease [*n* (%)]	1 (0.59)	1 (1.10)	#	>0.99
Chronic hypertension [*n* (%)]	9 (5.29)	9 (9.89)	1.950	0.163
Pre-gestational diabetes mellitus [*n* (%)]	0 (0)	4 (4.40)	#	0.014
Gestational diabetes mellitus [*n* (%)]	47 (27.65)	23 (25.27)	0.170	0.680
Other medicines during pregnancy			
Prophylactic anticoagulation [*n* (%)]	152 (89.41)	42 (46.15)	58.128	<0.001
Anti-inflammatory or immunosuppressive [*n* (%)]	81 (47.65)	13 (14.29)	28.627	<0.001

^#^Fisher exact probability method.

The medication regimen for HCQ used in this study was relatively uncomplicated, as the clinically recommended minimum effective dose was 6.5 mg/kg/day based on ideal body weight. The dosage form of drugs commonly sold in the region and caution in pregnancy may have contributed to the simplified regimen. None of the patients received long-term high-dose (>400 mg QD) HCQ, and in most cases(78.82%, 134/170), HCQ was administered long-term at a dose of 400 mg QD (200 mg BID) from preconception or early pregnancy to delivery.

The HCQ-treated group had a significantly higher proportion of patients receiving prophylactic anticoagulation (low molecular heparin and/or aspirin) and immune anti-inflammatory medications (oral glucocorticoids and/or immunosuppressants) than the non-HCQ-treated group (89.41% vs. 46.15%, *p* < 0.001; 47.64% vs. 14.29%, *p* < 0.001). Additionally, the proportion of patients with a history of ≥2 times early pregnancy losses was significantly higher in the HCQ-treated group than in the non-treated group (38.24% vs. 21.98%, *p* = 0.008).

### 3.2. Overall effect of HCQ on pregnancy outcomes in the ANAs-positive population

Table 2 presents the results of univariate and multifactorial analyses of perinatal outcomes related to placental dysfunction in pregnant women with ANAs positivity.

**Table 2:** Analysis of hydroxychloroquine and pregnancy outcomes in the population with positive antinuclear antibody spectrum.

	Application of HCQ	Without HCQ	OR (95% CI), *P*	aOR 1 (95% CI), a*P*1	aOR 2 (95% CI), a*P*2
	170 (65.13%)	91 (34.87%)			
Mid- to late-term pregnancy loss [*n* (%)]	0 (0)	4 (4.40)	0 (0, inf), *p* = 0.993	0 (0, inf), *p* = 0.997	0 (0, inf), *p* = 0.998
Pre-eclampsia [*n* (%)]	13 (7.65)	23 (25.27)	0.24 (0.11, 0.50), *p* < 0.001	0.25 (0.10, 0.58), *p* = 0.001	(0.002, 1.056), *p* = 0.067
Early onset pre-eclampsia [*n* (%)]	2 (1.18)	11 (12.09)	0.09 (0.01, 0.33), *p* = 0.002	0.0 7 (0.01, 0.29), *p* = 0.001	0.15 (0.02, 0.79), *p* = 0.040
Gestational week of delivery (weeks)	38.43 ± 2.31	36.34 ± 5.45	2.09 (1.14, 3.04), *p* < 0.001	1.87 (0.98, 2.76), *p* < 0.001	2.11 (1.11, 3.10), *p* < 0.001
Birth weight (g)	3075.87 ± 603.91	2847.53 ± 773.73	228.34 (59.31, 397.36), *p* = 0.009	2 40.94 (61.40, 420.23), *p* = 0.009	23 3.05 (30.21, 435.88), *p* = 0.025
Small for gestational age [cases/total, %]	18/179 (10.06)	23/89 (25.84)	0.32 (0.16, 0.63), *p* = 0.001	0.2 7 (0.13, 0.56), *p* < 0.001	0.2 8 (0.12, 0.64), *p* = 0.003
Neonatal asphyxia [cases/total, %]	3/176 (1.70)	2/86 (2.33)	0.73 (0.12, 5.61), *p* = 0.731	0.38 (0.05, 3.28), *p* = 0.332	0.16(0.02,1.49), *p* = 0.090

Birth weight and small-for-gestational-age infants were analyzed for fetuses with a gestational age of ≥24 weeks; Apgar score analysis was for live-born fetuses at all gestational weeks. aOR1, adjusts for differences in demographic and clinical characteristics; aOR 2, adjusts for other medications during pregnancy based on differences in demographics and clinical characteristics.

Model-1 included variables such as primiparas, obesity, and pre-gestational diabetes mellitus, which are known risk factors for preeclampsia. These variables showed a significant difference between the HCQ group and the group without HCQ (Table 1). In addition, age was also included as an independent risk factor. Model-2 included all variables in model-1 and other medications taken during pregnancy, such as prophylactic anticoagulation, anti-inflammatory or immunosuppressive drugs.

Both univariate and multivariate analyses showed that the use of HCQ during pregnancy was associated with a significant decrease in the incidence of early-onset preeclampsia (1.18% vs. 12.09%, *p* = 0.040) and SGA (10.06% vs. 25.84%, *p* = 0.003), as well as an increase of birth weight (3075.87 ± 603.91 vs. 2847.53 ± 773.73 g, *p* = 0.025) and prolonged gestation (38.43 ± 2.31 vs. 36.34 ± 5.45 weeks, *p* < 0.001).

### 3.3. ANA titer stratification and demographic and clinical characteristics

Of the 261 pregnant patients with ANAs positivity, 70.99% (185/261) were positive for ANA and 29.11% (76/261) were negative for ANA but positive for other antibodies in the spectrum of ANA. The ANA-positive cases were further stratified according to ANA antibody titers, with 127 patients in the ANA weakly positive group and 58 patients in the ANA positive group. The demographic and clinical characteristics of these populations with different ANA titers were presented in Table 3.

**Table 3:** Demographic information and clinical characteristics of populations with different titers of antinuclear antibodies.

Antinuclear antibody titer stratification [cases (%)]	Weakly positive	Positive	*t/X^2^*	*P-*value
	127 (74.71)	58 (34.11)		
Demographic and medical history information
Age (years)	33.96 ± 3.64	33.70 ± 3.63	0.451	0.653
Gravity (times)	2.66 ± 1.23	2.33 ± 1.14	1.731	0.085
Primiparas [*n* (%)]	102 (80.31)	49 (84.48)	0.461	0.497
History of early pregnancy loss ≥2 times [*n* (%)]	41 (32.28)	10 (17.24)	4.512	0.034
Obesity [Example (%)]	9 (7.09)	0 (0)	4.320	0.059
History of pre-eclampsia [*n* (%)]	6 (4.72)	4 (6.90)	0.367	0.508
This pregnancy
Embryo transfer [*n* (%)]	26 (20.47)	8 (13.79)	1.184	0.277
Multiple pregnancy [*n* (%)]	5 (3.93)	4 (6.90)	0.754	0.385
Weight gain during pregnancy (*g*)	12.48 ± 4.63	11.46 ± 4.64	1.389	0.167
Combined kidney disease [*n* (%)]	2 (1.57)	0 (0)	0.923	1.000
Chronic hypertension [*n* (%)]	34 (26.77)	10 (17.24)	1.995	0.158
Pre-gestational diabetes mellitus [*n* (%)]	2 (1.57)	0 (0)	0.923	1.000
Gestational diabetes mellitus [*n* (%)]	7 (5.51)	4 (6.90)	0.137	0.743
Other medicines during pregnancy
Prophylactic anticoagulation [*n* (%)]	89 (70.08)	49 (84.48)	4.359	0.037
Low-dose aspirin [*n* (%)]	85 (66.93)	43 (74.14)	0.971	0.325
Low molecular weight heparin [*n* (%)]	67 (52.76)	34 (58.62)	0.552	0.457
Hydroxychloroquine [*n* (%)]	74 (58.27)	47 (81.03)	9.121	0.003
Anti-inflammatory or immunosuppressants [*n* (%)]	42 (33.07)	20 (34.48)	0.036	0.850
Oral glucocorticoids [*n* (%)]	41 (32.28)	20 (34.48)	0.087	0.768
Other immunosuppressants [*n* (%)]	7 (5.51)	2 (3.45)	0.366	0.722

Notably, the application of HCQ during pregnancy was significantly higher in the ANA-positive group than in the weakly positive group (81.03% vs. 58.27%, *p* = 0.003), as was the combined application of prophylactic anticoagulation (84.48% vs. 70.08%, *p* = 0.037). However, no significant difference was found between the two groups in terms of combined anti-inflammatory or immunosuppressive therapies (34.48% vs. 33.07%, *p* = 0.850).

### 3.4. Stratified analysis of HCQ application and pregnancy outcomes in the ANA-positive population

The present study investigated the efficacy of HCQ in improving pregnancy outcomes within specific subgroups, as stratified by ANA titer and presented in Table 4.

**Table 4:** Stratified analysis of hydroxychloroquine and pregnancy outcomes in antinuclear antibody positive population.

	Application of HCQ	Without HCQ	*P*	OR (95% CI)	*P* interaction	a*P1*	aOR (95% CI)	*P1* interaction	a*P2*	aOR2 (95% CI)	*P2* interaction
Mid- to late-term pregnancy loss [*n* (%)]					>0.99			>0.99			>0.99
Weakly positive (*n* = 127)	0 (0/74)	3.77 (2/53)	0.996	0 (0, inf)		0.999	0 (0, inf)		0.999	0 (0, inf)	
Positive (*n* = 58)	0 (0/47)	0 (0/11)	0.999	0 (0, inf)		0.999	0 (0, inf)		0.999	0 (0, inf)	
Pre-eclampsia [*n* (%)]					0.369			0.486			0.354
Weakly positive (*n* = 127)	6.76 (5/74)	28.30 (15/53)	0.002	0.18 (0.06, 0.51)		0.004	0.18 (0.05, 0.55)		0.056	0.25 (0.06, 0.99)	
Positive (*n* = 58)	2.13 (1/47)	27.27 (3/11)	0.019	0.06 (0.03, 0.51)		0.029	0.19 (0.03, 0.48)		0.222	0.07 (0.03, 2.96)	
Early onset pre-eclampsia [*n* (%)]					0.195			0.259			0.264
Weakly positive (*n* = 127)	1.35(1/74)	13.21 (7/53)	0.027	0.09 (0.05, 0.53)		0.019	0.07 (0.04, 0.46)		0.244	0.23 (0.01, 2.06)	
Positive (*n* = 58)	0(0/47)	0 (0/11)	0.996	0(0, inf)		0.996	0 (0, inf)		0.996	0 (0, inf)	
Gestational week of delivery (weeks)					0.067			0.047			0.038
Weakly positive (*n* = 127)	38.39 (2.65)	37.05 (4.15)	0.028	1.34 (0.16, 2.52)		0.050	1.23 (0.01, 2.45)		0.263	0.82 (−0.61, 2.25)	
Positive (*n* = 58)	38.29 (2.54)	34.48 (7.68)	0.006	3.81 (1.19, 6.42)		0.010	3.93 (1.04, 6.82)		0.029	3.77 (0.49, 7.05)	
Birth weight (g)					0.263			0.342			0.345
Weakly positive (*n* = 127)	3101.84(636.39)	2789.07(776.31)	0.013	312.77 (69.38,556.16)		0.019	307.73 (54.69, 560.77)		0.340	325.53 (28.83, 622.23)	
Positive (*n* = 58)	2916.08(686.27)	2917.78(866.97)	0.995	−1.70 (−507.60,504.21)		0.939	23.46 (−571.25, 618.18)		0.704	128.31 (−529.87, 786.48)	
Small for gestational age [cases/total, %]					0.001			0.001			0.001
Weakly positive (*n* = 127)	7.89 (6/76)	35.19 (19/54)	<0.001	0.16 (0.05, 0.41)		<0.001	0.14 (0.05, 0.38)		0.002	0.13 (0.03, 0.43)	
Positive (*n* = 58)	19.61 (10/51)	0 (0/9)	0.994	0 (0, inf)		0.993	0 (0, inf)		0.995	0 (0, inf)	
Neonatal asphyxia [cases/total, %]					0.280			0.515			0.790
Weakly positive (*n* = 127)	1.35 (1/74)	3.85 (2/52)	0.387	0.34 (0.02, 3.67)		0.260	0.23 (0.02, 2.95)		0.322	0.22 (0.01, 4.43)	
Positive (*n* = 58)	4.00 (2/50)	0 (0/8)	0.997	0 (0, inf)		0.998	0 (0, inf)		0.998	0 (0, inf)	

aOR1, adjusts for differences in demographic and clinical characteristics; aOR2, adjusts for other medications during pregnancy based on differences in demographics and clinical characteristics.

Stratified univariate analysis indicated that HCQ use in the ANA-positive group significantly reduced the incidence of preeclampsia (2.13% vs. 27.27%, *p* = 0.019) and prolonged gestation (38.29 ± 2.54 vs. 34.48 ± 7.68 weeks, *p* = 0.006). In the ANA-weakly positive group, HCQ significantly reduced the incidence of preeclampsia (6.76% vs. 28.30%, *p* = 0.002), early-onset preeclampsia (1.35% vs. 13.21%, *p* = 0.027), and SGA (7.89% vs. 35.19%, *P*<0.001). Furthermore, HCQ use prolonged gestation (38.39 ± 2.65 vs. 37.05 ± 4.15 weeks, *p* = 0.028) and increased birth weight (3101.84 ± 636.39 vs. 2789.07 ± 776.31 g, *p* = 0.013) in this group.

Multivariate regression analysis, adjusting for demographic information, clinical characteristic factors, and concomitant medication use, showed that HCQ significantly reduced the incidence of preeclampsia in both ANA-positive and ANA-weakly positive groups, significantly prolonged gestation weeks in the ANA-positive group, and significantly reduced the incidence of SGA in the ANA-weakly positive group.

Finally, univariate and multivariate interaction analyses demonstrated no significant difference in HCQ efficacy in reducing the incidence of preeclampsia between the two ANA subgroups.

## 4. Discussion

### 4.1. Antinuclear antibodies may be associated with adverse pregnancy outcomes

Autoantibodies are produced as a result of immune dysregulation or cross-reactivity between foreign antigens and self-components. These autoantibodies target self-antigens and can elicit pathological immune responses, resulting in tissue damage and dysfunction (10). ANA, an antibody that targets intracellular components, is highly sensitive and can be used for screening despite its relatively poor disease specificity. ANAs, also known as the ANA profile, is a collection of antibodies that target various components of eukaryotic cells, including nuclear components, nucleic acids, nuclear proteins, cytoplasmic components, cytoskeletons, and cytokinesis cycle proteins. ANA profile exhibits varying sensitivities and specificities for specific AID diagnoses and serves as an important diagnostic tool for AID-assisted diagnosis (1).

Physiological autoantibodies can also be generated by a normal immune response, hence low ANA titers typically lack clinical significance in the general population, while high ANA titers (≥3 times the cut-off) have been linked to AID, such as SLE and SS. A higher ANA titer indicates a stronger association with AID, but does not imply greater disease severity. Rather, the titers of specific antibodies against target antigens within the ANA profile may be correlated with disease activity (1, 11). Moreover, the present study conducted a stratified investigation of different ANA titers and revealed that univariate and multivariate interaction analyses between the two groups demonstrated no significant difference in the efficacy of HCQ in improving the incidence of preeclampsia in patients with different ANA titers (Table 4).

The prevalence of ANAs has been found to be notably higher in patients experiencing embryo transfer failure and recurrent miscarriage compared to the general population (2, 4). Furthermore, the presence of ANAs has been shown to significantly increase the incidence of placental dysfunction-related adverse pregnancy outcomes, such as preeclampsia, in affected individuals (12). While the exact mechanism by which ANAs contribute to pregnancy loss is not yet fully understood, it is thought to involve several factors, including interference with immune tolerance at the maternal-fetal interface (13), damage to endothelial function (14, 15), and the inhibition of RNA transcription through the presence of RNA-binding complexes within ANAs (16).

### 4.2. Management status of pregnant women with positive ANAs test results who do not satisfy the diagnostic criteria of aid

For patients who exhibit a positive ANAs profile without evidence of a diagnosed AID, the potential impact of autoantibodies on maternal-foetal outcomes is not fully understood. As per previous guidelines and consensus, monitoring for clinical signs without drug recommendations is recommended (5, 17).

In the present study, individuals with positive ANAs test results often receive empirical medication, or even multiple medications, during pregnancy. This high degree of medication heterogeneity is linked to a lack of standardized clinical management, where the overdiagnosis of AID is frequently observed in clinical practice, and a subset of these patients may not receive adequate attention (10).

This study showed that patients with a history of ≥2 early pregnancy losses in the HCQ group was significantly higher than that in without HCQ group (38.24% vs. 21.98%, *p* = 0.008), and the proportion of primiparas was higher (87.65% vs. 70.33%, *p* = 0.001). These results suggest the contribution of the adverse pregnancy outcomes history. In clinical practice, a history of adverse pregnancy outcomes, or even infertility and repeated transplant failure with assisted reproduction, can increase the economic and psychological pressure on patients and their families, leading to an urgent desire for pregnancy, and may also motivating patients to seek empirical or combined medications (18). Additionally, insufficient observation time for related symptoms and abnormal immune indicators, the lack of standardized follow-up all can affect the clinical diagnosis of AID.

The overdiagnosis of connective tissue disease based solely on laboratory indicators, without considering clinical signs and symptoms, is common in this population. In addition to being a waste of health and economic resources, overdiagnosis exposes patients to potential risks associated with unnecessary medications, as illustrated in Table 1. Among ANA-positive patients, the use of HCQ during pregnancy was more common in those also receiving anticoagulation therapy (89.41% vs. 46.15%, *p* < 0.001) and glucocorticoid or other immunosuppressive therapy (47.65% vs. 14.29%, *p* < 0.001).

### 4.3. Value of HCQ in improving pregnancy outcomes related to placental dysfunction in ANAs-positive population

Recent reports indicate that HCQ reduces adverse pregnancy outcomes (7, 19, 20), including preeclampsia, in individuals with AID such as SLE (21), UCTD (22), and APS (23). Additionally, the safety of HCQ during pregnancy and lactation has been widely confirmed (24), indicating its potential as a drug option to improve placental function in individuals with autoimmune abnormalities.

Placental dysfunction can result in serious adverse maternal and fetal outcomes. However, the current drugs available for the prevention and treatment of these diseases have limited efficacy due to their single-targeted approach, including antispasmodic, antihypertensive, and circulatory improvement, and the limitations of aspirin in the prevention of preeclampsia (25, 26). Thus, it is essential to combine drugs with different targeting mechanisms to prevent and treat preeclampsia in clinical practice. Implementing multilevel preventive measures at each stage of maternal-fetal interface can further reduce the incidence of placental dysfunction diseases.

The widespread use of HCQ as a nonspecific immunoregulation drug in ANA-positive populations is primarily due to the recent focus on the inflammatory response at the maternal-fetal interface in autoimmune abnormalities (19). There is a broad suggestion of HCQ’s potential value in patients with abnormal autoimmune states to control immuno-inflammation. Our study’s findings further support the significance of HCQ in improving pregnancy outcomes related to placental dysfunction in ANA-positive populations.

### 4.4. Strengths and limitations

In this study, we aimed to address the issue of unstandardized diagnosis of AID during pregnancy and the potential overindication or unindicated use of immunosuppression drugs during pregnancy. We retrieved relevant data from the electronic medical record system of enrolled patients and rechecked the diagnosis in accordance with the recorded symptoms and physical and auxiliary examination results of the patients to ensure the accuracy of the study data.

However, this single-center retrospective study has some limitations. (i) Despite the use of stratified and multivariate regression analysis, the results may still be biased due to the limited sample size. (ii) The timeframe for the observation of relevant symptoms and abnormal laboratory indicators was insufficient due to the urgent desire of pregnancy in some cases. (iii) The review of the diagnostic process may have missed a very small proportion of autoimmunity that is still in a subclinical stage and was not identified and diagnosed. (iv) The study was based on obstetric hospital records, which excluded early pregnancy outcomes, such as biochemical pregnancy, graft failure, and early spontaneous abortion. (v) There is heterogeneity among patients, and the dosage and time frame of HCQ in some patients are not completely uniform.

### 4.5. Summary and outlook

In this study, we investigated the value of HCQ in improving placental dysfunction in pregnant women with ANAs positivity, based on the current empirical application of HCQ and the understanding of its pharmacological mechanism. We retrospectively analyzed cases from our hospital over the past 5 years and found a significantly higher incidence of adverse pregnancy outcomes in the ANAs-positive population, particularly related to complications of placental dysfunction such as preeclampsia. HCQ may be a promising pharmacological option for this population, with its multilevel immune anti-inflammatory mechanism and potential endothelial protective and thromboprophylactic effects, regardless of ANA titers. Clinical management of ANAs-positive patients often exhibits both overdiagnosis and underconcern, and a comprehensive judgment should be made by combining symptoms, signs, and medical histories to identify potential AID while avoiding overdiagnosis.

However, to validate our findings, a more in-depth and large-scale multi-center prospective cohort study will be conducted in the future. Additionally, investigating the mechanism of placental dysfunction caused by autoimmune abnormalities and the improvement of placental function by HCQ will be the focus of our next investigation.

## Data availability statement

The data that support the findings of this study are available from Third Hospital of Peking University but restrictions apply to the availability of these data, which were used under license for the current study, and so are not publicly available. However, data are available from the corresponding author on reasonable request and with permission of Peking University Third Hospital.

## Ethics statement

The studies involving human participants were reviewed and approved by Ethics Committee and Institutional Review Board of the Third Hospital of Peking University, People’s Republic of China (2022 No. 203-01). Written informed consent for participation was not required for this study in accordance with the national legislation and the institutional requirements.

## Author contributions

YW and SY contributed to conception and study design. SY wrote the main manuscript text. XZ, YL, and YM participated in collect, assemble, analyze, and interpret the data. All authors had full access to all of the data in the study and can take responsibility for the integrity of the data and the accuracy of the data analysis, read and approved the final manuscript.

## Funding

This study was supported by the National Key R&D Program of China (2022YFC2703504).

## Conflict of interest

The authors declare that the research was conducted in the absence of any commercial or financial relationships that could be construed as a potential conflict of interest.

## Publisher’s note

All claims expressed in this article are solely those of the authors and do not necessarily represent those of their affiliated organizations, or those of the publisher, the editors and the reviewers. Any product that may be evaluated in this article, or claim that may be made by its manufacturer, is not guaranteed or endorsed by the publisher.

## Abbreviations

HCQ: hydroxychloroquine
ANA: antinuclear antibody
ANAs: antinuclear antibody spectrum
AID: autoimmune diseases
SGA: small for gestational age
SLE: systemic lupus erythematosus
APS: antiphospholipid syndrome
SS: Sjögren’s syndrome
RA: rheumatoid arthritis
UCTD: undifferentiated connective tissue disease
